# Adverse childhood experiences and lower urinary tract symptoms in adolescence: the mediating effect of inflammation

**DOI:** 10.1093/ije/dyaf111

**Published:** 2025-07-02

**Authors:** Kimberley Burrows, Jon Heron, Gemma Hammerton, Ana L Goncalves Soares, Carol Joinson

**Affiliations:** Centre for Academic Child Health, Population Health Sciences, Bristol Medical School at the University of Bristol, Bristol, United Kingdom; Centre for Academic Mental Health, Population Health Sciences, Bristol Medical School at the University of Bristol, Bristol, United Kingdom; Medical Research Council Integrative Epidemiology Unit, Population Health Sciences, Bristol Medical School at the University of Bristol, Bristol, United Kingdom; Centre for Academic Mental Health, Population Health Sciences, Bristol Medical School at the University of Bristol, Bristol, United Kingdom; Medical Research Council Integrative Epidemiology Unit, Population Health Sciences, Bristol Medical School at the University of Bristol, Bristol, United Kingdom; Medical Research Council Integrative Epidemiology Unit, Population Health Sciences, Bristol Medical School at the University of Bristol, Bristol, United Kingdom; Centre for Academic Child Health, Population Health Sciences, Bristol Medical School at the University of Bristol, Bristol, United Kingdom

**Keywords:** adverse childhood experiences, ALSPAC, causal mediation, cohort study, CRP, g-formula, IL-6, incontinence, inflammation, lower urinary tract symptoms

## Abstract

**Background:**

There is evidence that adverse childhood experiences (ACEs) are associated with lower urinary tract symptoms (LUTS) in adulthood, but few studies have explored these associations in adolescence. Little is known about the biological mechanisms that could explain these associations.

**Methods:**

We used data from the Avon Longitudinal Study of Parents and Children (*n *=* *4745) on ACEs (from birth to age 8 years), LUTS at age 14 years [any urinary incontinence (UI), daytime and bedwetting, urgency, nocturia, frequent urination, voiding postponement, and low voiding volume], and inflammatory biomarkers interleukin-6 (IL-6) and C-reactive protein (CRP) measured at age 9 years. We examined associations between the summed ACE score and LUTS, and inflammation and LUTS. We then evaluated the mediating effects of IL-6 and CRP.

**Results:**

Higher ACE scores were associated with increased odds of LUTS, e.g. a one-unit increase in the ACE score was associated with an increased odds of any UI [odds ratio (OR) 1.16, 95% confidence interval (CI) 1.03–1.30]. Higher levels of IL-6 were associated with increased odds of LUTS, e.g. any UI (OR 1.24, 95% CI 1.05–1.47). There was weak evidence that the associations between ACE score and LUTS were mediated by IL-6 (e.g. any UI OR_natural_indirect_effect_ 1.03, 95% CI 1.00–1.06). There was no evidence that CRP was associated with LUTS or mediated the association between ACE score and LUTS.

**Conclusion:**

This study reports novel findings that point to inflammation as being a possible mechanism on the causal pathway from ACEs to LUTS. Early intervention is needed in childhood to prevent LUTS persisting into adolescence.

Key Messages

- We investigated whether adverse childhood experiences (ACEs) are associated with subsequent lower urinary tract symptoms (LUTS) mediated through inflammation.
- In this cohort study of 7475 young people, we report strong evidence that ACEs from birth to 8 years increase the odds of LUTS at age 14 years. We found weak evidence that this relationship is mediated by the inflammatory biomarker IL-6.
- We find that inflammation is a possible mechanism in the causal pathway from ACEs to LUTS, but further research is needed with more comprehensive measures of chronic inflammation.

## Introduction

Urinary incontinence (UI) is common in childhood, with bedwetting and daytime wetting affecting ∼15% and ∼8% of 7-year-olds, respectively [1, 2]. Although many cases of UI resolve during childhood, a significant proportion persist into adolescence, and new-onset cases also occur [3]. It is estimated that, at age 14 years, the prevalence of bedwetting and daytime wetting is 2.5% and 2.9%, respectively. Other lower urinary tract symptoms (LUTS) such as urgency and nocturia are also common (4.8% and 9.2%, respectively) [3]. Most cases of LUTS in children and young people are functional (non-organic) [4, 5] and can have an adverse effect on mental health [6, 7].

There is growing evidence that psychosocial factors in childhood, including stressful life events and adverse childhood experiences (ACEs), are associated with an increased risk of LUTS [8–11]. ACEs are traumatic experiences that include abuse, neglect, and household dysfunction. Although there is some evidence that certain types of adversity (e.g. sexual abuse [12]) are associated with an increased risk of LUTS, ACEs are often interrelated, making it difficult to isolate the effect of a specific ACE [13]. Instead, the total burden of exposure to ACEs during childhood is likely to be more important in determining the risk for subsequent LUTS. Previous research has mostly focused on women and found evidence of an association between ACEs and LUTS in a cohort study [14], case–control study [15], and convenience sample presenting to a urology clinic [10]. These studies are, however, limited by the retrospective recall of ACEs: small sample size [10, 15] and the lack of a control group without LUTS [10]. To the best of our knowledge, no prospective cohort studies have examined whether ACEs are associated with an increased risk of LUTS in adolescence.

There is robust evidence that ACEs lead to increased inflammation [16–18]. Inflammatory responses caused by exposure to chronic stress have been suggested as a mechanism through which ACEs increase the risk of LUTS [19]. The role of inflammation in LUTS is well established [20], with pro-inflammatory cytokines influencing the regulation of micturition pathways, as well as causing detrusor hypertrophy and afferent nerve hypersensitivity [19].

No cohort studies have examined inflammation as a mechanism linking ACEs to LUTS in a paediatric sample.

In the current study, based on data from the Avon Longitudinal Study of Parents and Children (ALSPAC), we examine prospective relationships between (i) ACEs (from birth to 8 years) and self-reported LUTS at age 14 years and (ii) inflammation at age 9 years and LUTS at age 14 years. We also examine evidence for a mediating effect of inflammation on the relationship between ACEs and LUTS.

## Methods

We used the Strengthening the Reporting of Observational Studies in Epidemiology cohort reporting guidelines (see Supplementary Material).

### Participants

ALSPAC is a prospective, population-based birth cohort study that recruited pregnant women resident in Avon, UK with expected dates of delivery between 1 April 1991 and 31 December 1992 [21, 22]. Of the initial 14 541 pregnancies, 13 988 children were alive a 1 year of age. Further details are given in the Supplementary text.

**Figure 1.**
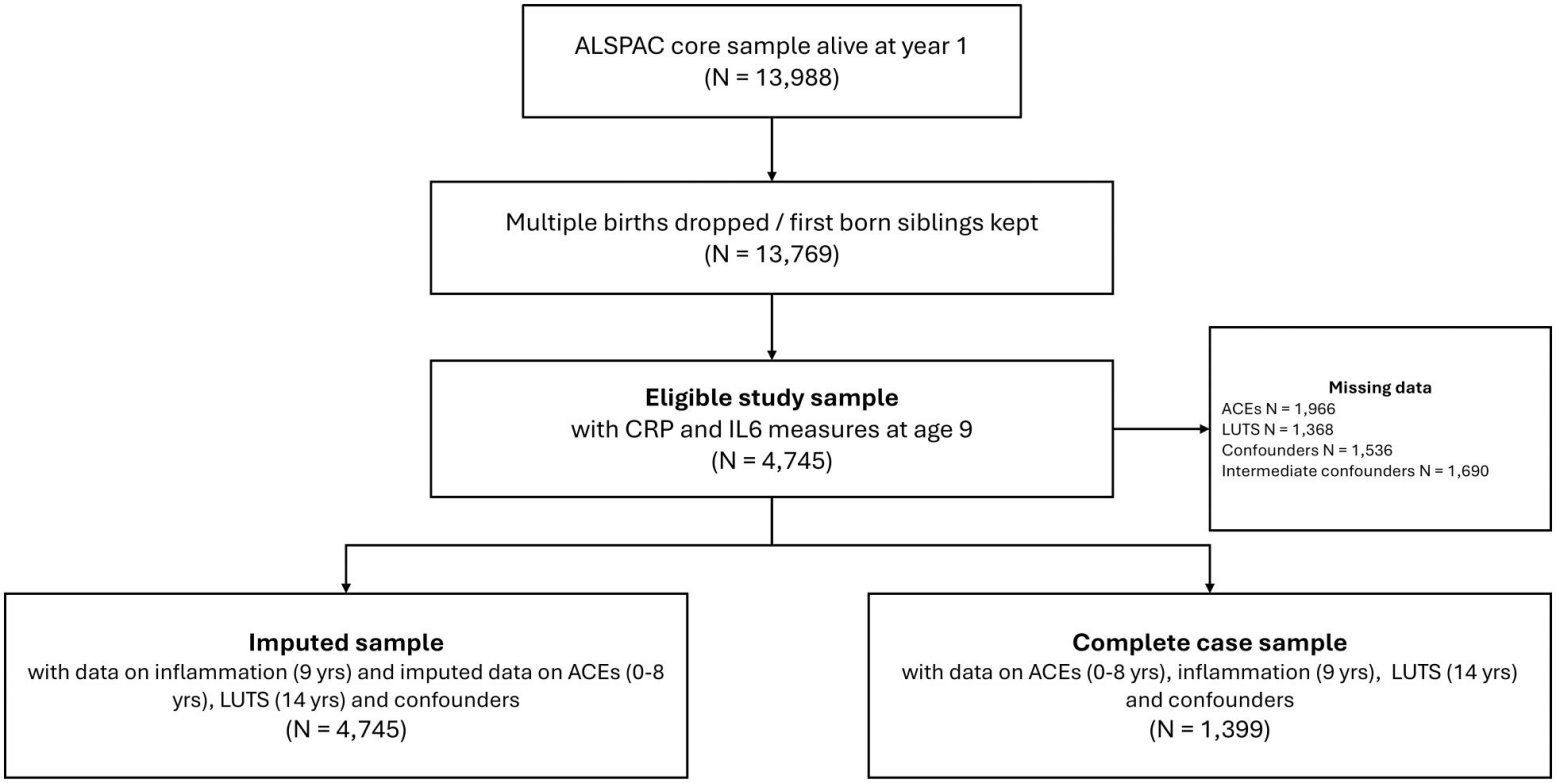
Flow chart of inclusion and exclusion of ALSPAC participants in the study sample.

The eligible sample included children who attended the ‘Focus@9’ clinic at a mean age of 9.9 years (SD 0.32; hereafter referred to as age 9 years) and provided a blood sample that was assayed for interleukin-6 (IL-6) and C-reactive protein (CRP) (*n = *4745). Complete data for inflammatory biomarkers, ACEs, LUTS, and confounders were available for 1399 children (Figure 1).

### Outcomes: LUTS

LUTS were assessed at age 14 years [mean (SD) 13.9 (0.14) years] via a self-report questionnaire [3]. Briefly, the questionnaire asked about the presence and frequency of LUTS within the past 2 weeks including daytime wetting, bedwetting, urgency, frequent urination, low voiding volume, voiding postponement, and nocturia. We additionally derived a variable indicating the presence/absence of any UI (either daytime and/or bedwetting) (Supplementary Table S1).

### Exposure: ACEs

Mothers, partners, and children were asked 254 questions across 20 time points about the child’s exposure (experienced vs. not experienced) to ACEs from birth up to age 8 years. The ACE score represents the overall burden of ACEs and is the summed score (scale of 0 to 10) of the 10 ‘classical’ ACEs: child sexual, physical, or emotional abuse; emotional neglect; substance abuse by the parents; parental mental illness or suicide attempt; violence between the parents; parental separation; bullying; and parental criminal conviction [23] (Supplementary Table S2). See Supplementary text for further information about the derivation of ACEs.

### Inflammatory biomarkers: IL-6 and CRP

We explored the mediating effects of two biomarkers of inflammation—IL-6 and CRP—measured by using clinical chemistry on non-fasted blood samples taken when the study children were aged 9 years [24]. Blood samples were immediately spun and frozen at –80°C. The biomarkers were assayed after a median of 7.5 years in storage with no previous freeze–thaw cycles. IL-6 (pg/ml) was measured by using enzyme-linked immunosorbent assay (R&D Systems) and high-sensitivity CRP (mg/L) was measured by using automated particle-enhanced immunoturbidimetric assay (Roche).

### Baseline and intermediate confounders

We adjusted for baseline confounders (measured antenatally or at birth) of each of the mediating pathways (exposure–outcome, exposure–mediator, and mediator–outcome) including maternal education, housing tenure, housing crowding, marital status, maternal smoking during pregnancy, maternal age at birth of child, parity, ethnicity, birthweight, gestational age, and sex assigned at birth. We also adjusted for child developmental delay (measured at 18 months). We adjusted for intermediate confounders at age 8 years that may be caused by ACEs and contribute to inflammation and LUTS (in addition to confounding the mediator–outcome associations). These included body mass index (BMI), constipation, and emotional and behavioural problems. We also adjusted for age at blood collection for the inflammatory biomarkers.

Details on the coding of the confounders are given in Supplementary Table S3.

### Statistical analysis

We used univariable and multivariable logistic regression to examine the relationships between (i) ACE score and LUTS and (ii) inflammatory biomarkers and LUTS, and linear regression to confirm previous ALSPAC results for ACE score and the inflammatory biomarkers. IL-6 and CRP measures were natural logarithmically transformed due to their skewed distribution.

**Figure 2.**
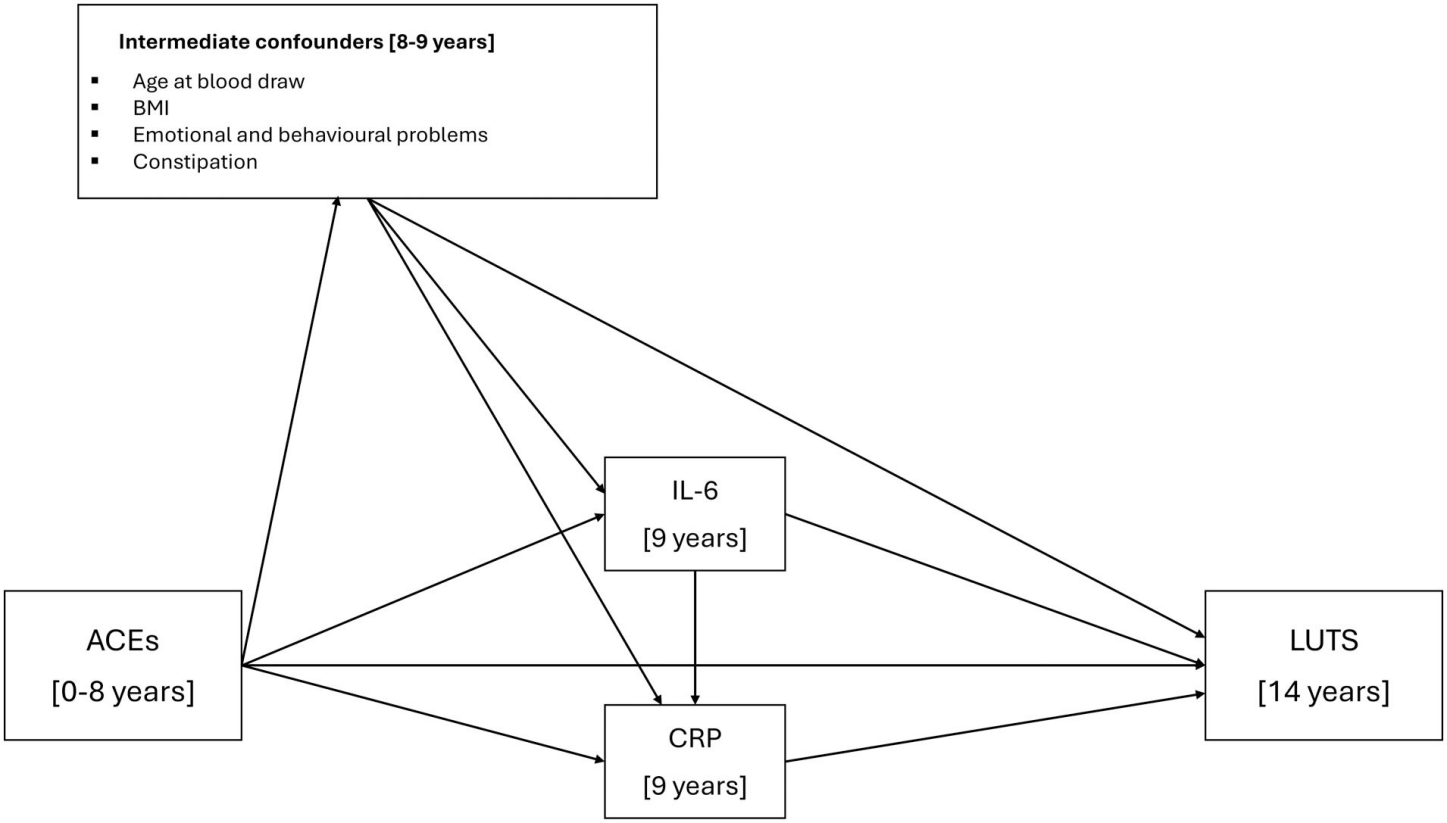
DAG illustrating hypothesized causal relationships between the exposures, mediators, and outcomes. DAG of the mediation model. Each intermediate confounding path (age at blood draw, child BMI, emotional and behavioural problems, and constipation) was specified separately but they are grouped together for clarity. For simplicity, baseline confounders are omitted from the DAG, as all confounders are proposed to cause all other model variables. DAG, directed acyclic graph.

We used the *gformula* (parametric g-computation formula [25]) command in Stata to examine the mediating effect of the inflammatory biomarkers on the association between ACE score and LUTS (see Figure 2 and Supplementary text for further information). G-formula is based on the counterfactual framework and allows the decomposition of effects into the total causal effect (TCE), the natural indirect effect (NIE) (via the mediators), and the natural direct effect (NDE) (not via the mediators). G-formula can also estimate marginal odds ratios (ORs) for mediation effects with a binary outcome whilst incorporating intermediate confounders. We used g-formula to decompose the indirect effects through IL-6 and CRP by using two models (see Supplementary Figure S1). For Model 1, we specified IL-6 as the mediator whilst omitting CRP because we hypothesized that CRP is on the causal pathway from IL-6 to LUTS; for Model 2, we specified CRP as the mediator and IL-6 as an intermediate confounder, preserving the biological pathway between the two biomarkers. We used a Monte Carlo sample size of 100 000 and we estimated normal-based 95% confidence intervals (CIs) by using standard errors from 500 bootstrap resamples. The proportion mediated (PM; %) was calculated as [OR_NDE_ (OR_NIE_ − 1)]/[OR_NDE_ × OR_NIE_ − 1] × 100 [26]; however, this is only interpretable when the TCE, NIE, and NDE have the same direction of effect.

Analyses were conducted by using Stata version 17 [27] and R version 4.3.0 [28].

### Missing data

We used multivariate imputation by chained equations under the missing at random assumption to impute missing data [29] in the individual ACEs, LUTS, and confounders up to the sample with complete data on IL-6 and CRP (*n = *4745). We also included auxiliary variables that are considered predictive of the missing values (Supplementary Table S4). Missing data were imputed separately in males and females before appending the two datasets together. For both males and females, 80 imputed datasets with 50 iterations were created by using the mice package (version 3.16.0) in R. Estimates were then combined by using Rubin’s rules [30]. See Supplementary text for further information.

## Results

Supplementary Tables S5–S7 show the descriptive statistics of study variables for the imputed and complete case samples. Any UI was reported by 5.3% of adolescents, with 4.6% and 4.5% of adolescents experiencing daytime wetting and bedwetting, respectively. The most common LUTS were voiding postponement (15.6%) and nocturia (10.5%), while a high frequency of urination was the least common (4.3%). Further details of the study sample and variables are given in the Supplementary text (‘Results: description of sample’).

### Associations between ACEs and inflammation

There was evidence that a higher ACE score was associated with a higher level of IL-6 (expressed as the percentage change in IL-6 per unit increase in ACE score) [1.03 (3%), 95% CI 1.01–1.05 (adjusted); Supplementary Table S8] but not CRP. There was strong evidence of an association between IL-6 and CRP [1.78 (78%), 95% CI 1.72–1.84 (adjusted); Supplementary Table S8].

### Associations between ACEs and LUTS

Higher ACE scores were associated with increased odds of LUTS, e.g. a one-unit increase in the ACE score was associated with an increase in the odds of any UI [OR 1.16, 95% CI 1.03–1.30 (adjusted)]. There were similar associations with all other LUTS; however, the 95% CIs for daytime wetting and bedwetting crossed the null (Table 1). The direction of association for each association was concordant in the complete case analysis (see Supplementary Table S9).

**Table 1.** Associations between ACE score and LUTS in the imputed data sample (*n *=* *4745)

Outcome	Univariable			Multivariable		
	OR	95% CI	*P*-value	OR	95% CI	*P*-value
UI (any)	1.20	1.08, 1.34	<0.001	1.16	1.03, 1.30	0.015
Daytime wetting	1.18	1.03, 1.35	0.020	1.15	0.98, 1.34	0.081
Bedwetting	1.21	1.05, 1.40	0.010	1.17	0.99, 1.37	0.063
Urgency	1.23	1.11, 1.38	<0.001	1.19	1.06, 1.34	0.004
Nocturia	1.21	1.12, 1.32	<0.001	1.15	1.05, 1.26	0.004
Frequency	1.33	1.19, 1.50	<0.001	1.29	1.12, 1.48	<0.001
Voiding postponement	1.13	1.05, 1.22	<0.001	1.10	1.02, 1.19	0.019
Voiding volume	1.19	1.06, 1.34	0.003	1.15	1.00, 1.31	0.045

Multivariable models adjusted for baseline confounders (sex, birthweight, gestational age, ethnicity, child developmental delay, maternal age, smoking during pregnancy, parity, maternal education, marital status, house tenure, and crowding). Analysis performed in 80 imputed datasets.

### Associations between inflammation and LUTS

A one-unit increase in log IL-6 was associated with an increase in the odds of any UI, daytime wetting, and nocturia in the adjusted models (Table 2). There was weak evidence that IL-6 was associated with reduced odds of voiding postponement (OR 0.89, 95% CI 0.79–1.00). The 95% CIs for associations of IL-6 with the other LUTS crossed the null. The associations were similar in the complete case analysis (see Supplementary Table S10). All 95% CIs crossed the null in the analysis of the association between CRP and LUTS.

**Table 2.** Associations between inflammatory biomarkers and LUTS in the imputed data sample (*n *=* *4745)

Outcome	Univariable			Multivariable		
	OR	95% CI	*P*-value	OR	95% CI	*P*-value
**Exposure: logIL-6 (pg/ml)**						
UI (any)	1.29	1.11, 1.51	0.001	1.24	1.05, 1.47	0.011
Daytime wetting	1.50	1.21, 1.86	<0.001	1.41	1.11, 1.79	0.005
Bedwetting	1.14	0.92, 1.42	0.232	1.11	0.89, 1.39	0.336
Urgency	1.18	0.99, 1.40	0.058	1.10	0.92, 1.32	0.311
Nocturia	1.24	1.09, 1.42	0.001	1.16	1.01, 1.34	0.039
Frequency	1.24	0.99, 1.56	0.060	1.15	0.90, 1.46	0.270
Voiding postponement	0.94	0.85, 1.05	0.293	0.89	0.79, 1.00	0.055
Voiding volume	1.07	0.89, 1.29	0.489	1.00	0.82, 1.22	0.983
**Exposure: logCRP (mg/L)**						
UI (any)	1.04	0.93, 1.17	0.449	1.03	0.91, 1.17	0.694
Daytime wetting	1.11	0.96, 1.30	0.166	1.06	0.89, 1.27	0.516
Bedwetting	0.99	0.85, 1.16	0.927	1.02	0.86, 1.21	0.839
Urgency	1.02	0.89, 1.15	0.814	0.96	0.84, 1.11	0.590
Nocturia	1.10	1.01, 1.21	0.034	1.06	0.95, 1.17	0.292
Frequency	1.12	0.96, 1.30	0.153	1.10	0.93, 1.30	0.273
Voiding postponement	0.99	0.91, 1.07	0.743	0.96	0.88, 1.04	0.323
Voiding volume	1.03	0.90, 1.17	0.694	1.01	0.87, 1.17	0.930

Multivariable models adjusted for baseline confounders (sex, birthweight, gestational age, ethnicity, child developmental delay, maternal age, smoking during pregnancy, parity, maternal education, marital status, house tenure, and crowding), intermediate confounders (age at blood draw, child BMI, emotional and behavioural problems, and constipation), and adverse childhood experience score. Analysis performed in 80 imputed datasets; log is the natural logarithm.

### Mediation results

Table 3 shows the TCE, NIE, NDE, and PM for Model 1 (mediation through IL-6). There was weak evidence that the association between ACE score and any UI was mediated by IL-6 (OR_NIE_ 1.03, 95% CI 1.00–1.06, PM 21%). There was also weak evidence for a mediating effect of IL-6 between ACE score and daytime wetting (OR_NIE_ 1.06, 95% CI 1.01–1.10, PM 45%), bedwetting (OR_NIE_ 1.04, 95% CI 1.00–1.08, PM 31%), urgency (OR_NIE_ 1.03, 95% CI 1.00–1.07, PM 19%), and frequency (OR_NIE_ 1.04, 95% CI 1.00–1.08, PM 18%). There was no evidence of mediating effects of IL-6 on the association between ACE score and nocturia, voiding postponement, and voiding volume.

**Table 3.** Model 1 results for the mediation of ACE score and LUTS via IL-6 in the imputed data sample (*n *=* *4745)

Outcome	Total causal effect		Natural indirect effect		Natural direct effect		PM (%)
	OR	95% CI	OR	95% CI	OR	95% CI	
UI (any)	1.17	1.04, 1.31	1.03	1.00, 1.06	1.13	1.01, 1.28	21
Daytime wetting	1.14	0.98, 1.33	1.06	1.01, 1.10	1.08	0.92, 1.27	45
Bedwetting	1.15	0.98, 1.35	1.04	1.00, 1.08	1.10	0.93, 1.31	31
Urgency	1.19	1.06, 1.33	1.03	1.00, 1.07	1.15	1.02, 1.30	19
Nocturia	1.15	1.05, 1.25	1.01	0.99, 1.04	1.13	1.03, 1.24	8
Frequency	1.27	1.11, 1.45	1.04	1.00, 1.08	1.22	1.06, 1.40	18
Voiding postponement	1.09	1.01, 1.18	0.99	0.97, 1.02	1.10	1.01, 1.19	NA
Voiding volume	1.14	1.00, 1.31	1.03	0.99, 1.07	1.11	0.97, 1.28	23

OR, per one-unit increase in adverse childhood experience score; NA, not applicable. Mediation models were fitted with IL-6 as the mediator of interest; age at blood draw, child BMI, emotional and behavioural problems, and constipation were considered intermediate confounders. All paths were adjusted for baseline confounders: sex, birthweight, gestational age, ethnicity, child developmental delay, maternal age, smoking during pregnancy, parity, maternal education, marital status, house tenure, and crowding. Analysis performed in 80 imputed datasets.

There was no evidence of a mediating effect of CRP (Model 2) on the associations between ACE score and LUTS (see Supplementary Table S11) after accounting for IL-6 as an intermediate confounder.

The results for the complete case analysis of the mediating effects of IL-6 (Model 1) and CRP (Model 2) on the association between ACE score and LUTS are provided in Supplementary Tables S12 and S13, respectively. The directions of effect and overall findings were similar.

## Discussion

Our study is the first to assess prospective associations between ACEs and LUTS in adolescence. An increased burden of ACEs was associated with an increase in the odds of LUTS in adolescence. Our findings are consistent with earlier research that has reported an enduring effect of the total burden of ACEs on the risk of LUTS in adults [10, 14, 15]. Another novel aspect of our study is the examination of the prospective relationship between biomarkers of inflammation in childhood and LUTS in adolescence. We found that higher levels of the inflammatory biomarker IL-6 (but not CRP) at age 9 years were associated with increase odds of subsequent LUTS including any UI, daytime wetting, and nocturia. This is also the first cohort study to examine the mediating effect of inflammation as a possible biological mechanism that could underlie the relationship between ACEs and subsequent LUTS. We found weak evidence of a mediating effect of IL-6 on the relationship between ACEs and LUTS.

### Strengths and limitations

Strengths of our study include the use of data from a large population‐based birth cohort, the prospective design, the availability of data on a range of self-reported LUTS in adolescence, and adjustment for a comprehensive set of confounders. ACEs were prospectively assessed at multiple time points by multiple informants (mothers, partners, and children) compared with previous research that has relied on the retrospective recall of ACEs. We assessed the total burden of ACEs because it is difficult to isolate the effect of a specific ACE [13]. We used adolescents’ self-reports of LUTS and did not restrict our analysis to those who met clinical diagnostic criteria. The study, therefore, provides evidence that ACEs are prospectively associated with LUTS in adolescents in the community.

Limitations should be considered when interpreting the current findings. Nonresponse and loss to follow‐up occur more frequently among individuals who are exposed to socioeconomic disadvantage and ACEs [31]. The complete case sample had a lower proportion of participants with socioeconomic disadvantage and ACEs than the wider ALSPAC cohort, which may have limited the internal validity of our findings. We therefore used multiple imputations to address possible bias due to missing data and report all results as recommended by Sterne *et al*. [32].

The ALSPAC cohort is predominantly White and affluent [21, 22] and hence we are unable to generalize our results to minority ethnic groups and less affluent populations.

Inflammatory biomarkers were measured in non-fasting bloods at a single time point; therefore, we do not have robust data on the consistency of systemic levels of IL-6 and CRP within individuals over time. This is a potential limitation given the effects of age and puberty status on inflammation levels during childhood and adolescence [33]. It is also possible that non-fasting blood samples of inflammatory biomarkers exhibit diurnal variation, resulting in increased measurement error [34].

Due to multiple testing, there is a possibility of an increase in type 1 errors. We do not, however, rely on *P*-value thresholds to determine whether the results are statistically significant. Consistently with the recommended approach, we present effect estimates with their 95% CIs in addition to the *P*-values [35, 36].

### Potential mechanisms that could explain the findings

ACEs have previously been described as getting ‘under the skin’ and inducing ‘physiological changes’ [37] that can lead to an increased risk of chronic health problems [38]. The physiological changes include an increased risk of systemic inflammation [39], which, in turn, has been implicated as a risk factor for LUTS [20]. There is evidence from animal models that exposure to chronic stress and early-life adversity can have an impact on lower urinary tract function due to functional and structural changes in the bladder, especially in the detrusor muscle and urothelium, and can lead to hypersensitivity of the afferent nerves in the bladder [19].

We cannot rule out reverse causality because persistent incontinence in children places a considerable burden on their families [40] and it is not uncommon for parents to adopt a negative coping style [41]. Therefore, the child’s incontinence could be a cause, not a consequence, of ACEs. In terms of the direction of the relationship between inflammation and LUTS, previous evidence suggests that pro-inflammatory cytokines influence the regulation of micturition pathways, leading to LUTS [19]. Accumulation of residual urine can sometimes be a consequence of LUTS, leading to an increased risk of urinary tract infection (UTI) [42] and a temporary increase in inflammation [19]. Studies that examine inflammatory biomarker levels often exclude children with an infection that coincides with the time of blood collection because levels of IL-6 and CRP increase in response to acute infection but return to baseline afterwards. However, current infections could include UTIs and excluding these individuals could result in collider bias because LUTS are outcomes in the models [43]. Consequently, we did not exclude individuals that reported any recent infection at blood sampling (*n = *446; 9.4%) or those with a CRP level of >10 mg/L (*n = *57; 1.2%). A limitation of this is that some children will have temporarily inflated inflammation levels owing to other infection sources such as colds.

We found evidence that IL-6, but not CRP, mediates the association between ACE score and LUTS. This is contrary to our hypothesis given that IL-6 upregulates the synthesis of CRP and both have functional roles in immunity [44]. Despite their interrelationship, IL-6 and CRP have distinct signalling pathways as well as functional roles in absence of inflammation [45]. IL-6 is a pleiotropic cytokine operating through two distinct signalling pathways; classical and trans, the former of which is anti-inflammatory and results in the upregulation of CRP synthesis via signalling in hepatocytes [46]. The trans-signalling pathway is pro-inflammatory, acts upon all cells, and is associated with chronic inflammatory conditions [46, 47]. It is plausible that, mechanistically, our results reflect the effects of IL-6 trans-signalling on the risk of developing LUTS, independently of the classic signalling pathway that upregulates CRP levels, but further research is needed to confirm this. Future research could also explore the role of composite markers of inflammation rather than individual measures at single time points (e.g. DNA methylation scores and GlycA [48, 49]).

Our findings may also reflect measurement error, as blood samples at age 9 years were taken in a non-fasting state and therefore may have been subject to diurnal variation [34]. However, measurement error is likely to be random in relation to our outcomes.

### Conclusions

This study reports novel findings that IL-6 was associated with LUTS and provides weak evidence of a role of inflammation as a possible mechanism linking ACEs to LUTS in adolescence. It is important to note that IL6 and CRP provide only a limited measure of chronic inflammation and further research is needed in other samples and with a wider range of inflammatory biomarkers measured at repeated time points to more robustly test whether inflammatory processes mediate the effect of ACEs on LUTS. Further examination of biological, behavioural, and psychological mechanisms will provide a more complete understanding of causal pathways from adversity to LUTS. Evidence of mechanisms linking ACEs to LUTS could lead to the identification of novel translational targets for intervention and potential therapeutic advances in the treatment of LUTS. ACEs can be difficult to modify, so targeting the mechanisms could lead to secondary preventions aimed at reducing the risk of chronic LUTS.

Our findings should raise awareness amongst clinicians of the importance of screening for ACEs in children presenting with LUTS, as they could be contributing factors and can negatively affect treatment outcomes amongst children with LUTS [50].

## Ethics approval

Ethical approval for the study was obtained from the ALSPAC Ethics and Law Committee and the Local Research Ethics Committees. Informed consent for the use of data collected via questionnaires and clinics was obtained from participants following the recommendations of the ALSPAC Ethics and Law Committee at the time. Consent for biological samples has been collected in accordance with the Human Tissue Act (2004).

## Supplementary Material

dyaf111_Supplementary_Data

## Data Availability

The ALSPAC data underlying this article are available upon request from the ALSPAC Executive Committee for researchers who meet the criteria for access to confidential data (https://bristol.ac.uk/alspac/researchers/access/). The code to conduct these analyses can be found at https://github.com/burrowsk/ACEs-Inflamamtion-LUTS.
